# Target leakage and the use of diagnostic variables in diabetes prediction models

**DOI:** 10.1038/s41387-025-00396-5

**Published:** 2025-10-27

**Authors:** Melike Tombaz, Nico Pfeifer, Sabrina Ehnert, Andreas K. Nüssler

**Affiliations:** 1 Siegfried Weller Institute for Trauma Research, BG Unfallklinik Tübingen, University of Tübingen, Tübingen, Germany; 2 Institute for Bioinformatics and Medical Informatics, University of Tübingen, Tübingen, Germany

**Keywords:** Diagnosis, Risk factors

**Dear Editor**,

We read the article “Optimization of diabetes prediction methods based on combinatorial balancing algorithm” by Shao et al., 2024, with great interest [1]. The authors tackle a significant technical issue in biomedical data science: class imbalance in diabetes prediction models. By combining SMOTE with random under-sampling (RUS) and using Optuna for hyperparameter tuning of LightGBM, they enhance the ongoing efforts to boost model performance in imbalanced datasets.

However, we would like to raise a methodological concern in the training process that may impact the interpretation and clinical applicability of the findings. The authors have incorporated HbA1c levels and blood glucose values in the presented models as predictor variables for determining diabetes status. These parameters are integral to the standard clinical diagnostic criteria for diabetes [2]. Including them might cause target leakage, which occurs when a model is trained on input features that either directly influence or are closely linked to the result [3].

Such leakage can result in inflated accuracy and precision during evaluation while diminishing the model’s relevance for early detection or screening tasks. When features inherently linked to diagnosis are used in training, the model’s predictive validity becomes compromised [4]. This issue has previously been noted in other contexts as well, such as the use of anti-hypertensive drugs to predict hypertension [5].

While the study mainly targets class imbalance, incorporating diagnostic variables influences the model’s relevance. As previously mentioned, leakage distorts performance metrics and reduces generalizability in real-world scenarios [6]. Consequently, relying on HbA1c levels during the training phase can lead to artificially high performance metrics during evaluation; the model may excel in its predictive accuracy based on the training data, but may fail to effectively diagnose new patients in a real-world clinical setting.

In clinical machine learning applications, particularly for early screening and risk assessment, developing models that account for real-world limitations is crucial. HbA1c levels alone can often provide a diabetes diagnosis, eliminating the need for a predictive model. Thus, incorporating diagnostic variables like HbA1c in model training reduces the model’s applicability in pre-diagnostic scenarios. The primary aim in these cases is to identify individuals at higher risk before definitive diagnostic information becomes available.

It is important to exclude diagnostic criteria from the training process to improve clinical applicability and avoid misleading performance estimates, especially when the goal is to identify patients at increased risk rather than confirm existing disease. Future studies aiming to predict the risk of diabetes mellitus may benefit from a more cautious feature selection strategy to ensure the development of clinically meaningful and generalizable models.
